# Cholera in travellers: a systematic review

**DOI:** 10.1093/jtm/taz085

**Published:** 2019-12-04

**Authors:** Bradley A Connor, Richard Dawood, Mark S Riddle, Davidson H Hamer

**Affiliations:** 1 Weill Cornell Medical College and the New York Center for Travel and Tropical Medicine, New York, NY, USA; 2 Fleet Street Travel Clinic, London EC4Y 1AA, UK; 3 University of Nevada, Reno School of Medicine, Reno, NV, 89557, USA; 4 Department of Global Health, Boston University School of Public Health, Boston, MA, USA; 5 Section of Infectious Diseases, Department of Medicine, Boston Medical Center, Boston, MA, USA

**Keywords:** Serogroup O139, Vibrio cholera, Serogroup 01, vibriocidal antibody, Cholera vaccine, Cholera risk

## Abstract

Exposure to cholera is a risk for individuals and groups travelling to endemic areas, and the bacteria can be imported to cholera-free countries by returning travellers. This systematic review of the literature describes the circumstances in which cholera infection can occur in travellers and considers the possible value of the cholera vaccine for prevention in travellers. PubMed and EMBASE were searched for case reports of cholera or diarrhoea among travellers, with date limits of 1 January 1990–30 April 2018. Search results were screened to exclude the following articles: diarrhoea not caused by cholera, cholera in animals, intentional cholera infection in humans, non-English articles and publications on epidemics that did not report clinical details of individual cases and publications of cases pre-dating 1990. Articles were reviewed through descriptive analytic methods and information summarized. We identified 156 cases of cholera imported as a consequence of travel, and these were reviewed for type of traveller, source country, serogroup of cholera, treatment and outcomes. The case reports retrieved in the search did not report consistent levels of detail, making it difficult to synthesize data across reports and draw firm conclusions from the data. This clinical review sheds light on the paucity of actionable published data regarding the risk of cholera in travellers and identifies a number of gaps that should drive additional effort. Further information is needed to better inform evidence-based disease prevention strategies, including vaccination for travellers visiting areas of cholera risk. Modifications to current vaccination recommendations to include or exclude current or additional traveller populations may be considered as additional risk data become available. The protocol for this systematic review is registered with PROSPERO (registration number: 122797).

## Introduction

Cholera, an acute, secretory diarrhoeal disease caused by toxigenic strains of Gram-negative bacterium *Vibrio cholerae* (O1 and O139 serogroups), is spread through contaminated food and water.^1^^,^^2^ Improved sanitation and access to safe water have largely eliminated indigenous cholera in high-income countries, but cholera remains a problem in lower income countries, where adequate sanitation and safe water are not widely available and large epidemics can occur. Cholera is endemic in at least 47 countries^2^^,^^3^ but this number is dynamic as affected countries, as listed on the Centers for Disease Control and Prevention (CDC) website, frequently change.^1^ It is believed that 1.4 billion people are at risk from cholera in endemic countries, with an estimated 1.2 million cases annually.^4^ Cholera usually manifests itself as diarrhoea, though not usually as ‘cholera gravis’ (i.e. profuse watery diarrhoea that results in death if not rapidly treated).^5^ The World Health Organization (WHO) has estimated that officially-reported cases represent only 5–10% of the true number of cases.^6^ Countries with significant endemic seasonal transmission still do not publicly report cases of cholera, while countries with outbreaks continue to report cases and deaths due to ‘acute watery diarrhoea’.^4^ Considering the insufficient number of surveillance studies, efforts have been made to estimate the cholera disease burden by using modelling approaches.^3^ Notably, the number of cholera cases in the USA is estimated to be ~33 times higher than those diagnosed^7^ but this may be primarily of academic interest as the majority of these are mild cases with limited public health import. There have also been recent reports of an underappreciated asymptomatic carrier state, the prevalence of which may be as high as 3–100 asymptomatic individuals for every clinical case.^8^

Exposure to cholera is a possible risk for individuals and groups traveling to endemic countries (such as tourists, business travellers, those engaging in humanitarian, medical or missionary work, or the military), with the degree of risk varying according to specific areas visited and the duration of stay.^1^ In addition, cholera can be imported from areas where it is endemic or epidemic to cholera-free countries.^2^^,^^9^ In 2017, the WHO reported 675 cases of imported cholera, with 12 of these being in North America.^4^ However, it is widely recognized that there may be widespread under-reporting and under-diagnosis of cholera globally, due to economic, social and political disincentives, inadequate investigation or lack of diligence.^3^^,^^10^ Differentiation of cholera from other diarrhoeal diseases on clinical grounds is often difficult^9^^,^^11^; poor laboratory resources and epidemiological surveillance in endemic regions also hinder diagnosis, and within an outbreak setting, not all specimens may be tested.^12^

Cholera is a rare disease among travellers from non-endemic to endemic areas, with an estimated risk of 0.2 cases per 100 000 European and North American travellers.^13^ In healthy adults travelling to endemic areas, cholera is effectively treated as, and not distinguished from, other causes of acute watery travellers’ diarrhoea (TD) and is under-reported as a specific cause of illness.^9^^,^^14^^,^^15^ Thus, the burden of cholera in travellers is not well understood because most cases are not reported. Effective antibiotic treatment can shorten the duration of illness and reduce shedding of the infectious agent.^15^^,^^16^

Current recommendations for TD, including probable and confirmed cholera, occurring in endemic or outbreak settings are for rehydration therapy and antibiotic treatment (with or without loperamide) for all secretory (watery) cases.^14^^,^^15^^,^^17^

The quest for a cholera vaccine dates back to Louis Pasteur’s early work on vaccines in the 1870s and gained substantial attention after the current cholera pandemic began in 1961.^5^^,^^14^ In modern times, the whole-cell killed parenteral vaccines for international travel came into wider use during the years that followed, both on its own and in combination with typhoid vaccine.^19^ In an effort to control the spread of cholera across international boundaries (and shortly after the success of vaccination in controlling the spread of smallpox), the WHO introduced mandatory cholera vaccination in 1969.^20^ However, it rapidly became apparent that the parenteral vaccines then in use were not effective at controlling the spread of cholera, and it also became clear that the prevailing serogroups were causing a milder illness than ‘classic’ cholera. In 1973, the World Health Assembly deleted from the International Health Regulations the requirement for presentation of a cholera vaccination certificate,^21^ and the WHO recommended that its partner countries no longer require cholera vaccination for entry of travellers.

However, given the persistent threat of cholera among resource poor populations of the world, and the growth in global travel to regions far and wide, there has been a renewed focus since the 1990s on developing safer and more effective oral cholera vaccines. Vaccines for cholera are now widely available, including for travellers, though it should be noted that these vaccines have limited effectiveness against the El Tor biotype of *V. cholerae*. A better understanding of the current epidemiology of cholera is needed to help evaluate the role of vaccination beyond the endemic populations at highest risk. We carried out a systematic review of the literature to provide information on the circumstances in which cholera infection has been reported in travellers and to consider the utility of vaccination for prevention of cholera in travellers.

## Methods

### Data sources and searches

PubMed and EMBASE were searched using the search terms such as (cholera^*^ OR diarrh^*^ in Title) AND (Imported OR travel^*^ OR touris^*^ OR migrant^*^ OR immigrant^*^ OR migrat^*^ OR immigrat^*^ OR refugee^*^ OR military OR soldier^*^ OR troops OR army OR armies OR war OR forces in All Fields) with date limits of 1 January 1990–30 April 2018. Cases mentioned in reviews but not found in the searches were retrieved by hand.

Apart from abstracts listed in EMBASE, grey literature (e.g. government resources and congress publications) was not included, and duplicate articles were removed.

### Study selection

The search results were screened by a non-blinded reviewer to exclude the articles or publications on: diarrhoea not caused by cholera, cases caused by non-O1 or non-O139 serogroups of *V. cholerae*, cholera in animals, intentional cholera infection in humans (e.g. challenge studies, vaccine studies and microbiological studies), reporting epidemics where clinical details of individual cases are not reported, non-English articles (if there was sufficient information in an English abstract, they were included) and those including cases pre-dating 1990.

### Data extraction and quality assessment

Possible articles of interest were retrieved and reviewed, and the accuracy of extracted data was assessed by an independent reviewer. Dual validation was not performed as this was a search for case report data, rather than a quantitative meta-analysis.

### Data synthesis and analysis

Description of study type (case-control, cohort case series), year of publication, country of origin, country of destination, duration of travel (mean or median) and population type (business, casual, military, etc.) were reviewed through descriptive analytic methods, and information about the number of cases, serotype and circumstances of the infection were summarized.

For descriptive purposes, cases were grouped according to the following parameters: caused by food but travel-associated, serotype involved, treatment (if reported), duration of illness and illness outcome (complete recovery, death, secondary transmission).

‘Food’ was not included in the search strategy, and therefore, the focus of this review is on travel-associated cholera rather than cases resulting from consumption of imported contaminated food.

The details of this systematic review have been registered on the PROSPERO site, registration number 122797.

### Role of the funding source

Independent editorial support was provided by Elements Communications Ltd and was funded by Emergent BioSolutions. Emergent BioSolutions was not involved with the design, analysis, interpretation of data, writing, editing or approval for publication.

## Results

### Study characteristics

**Figure 1:**
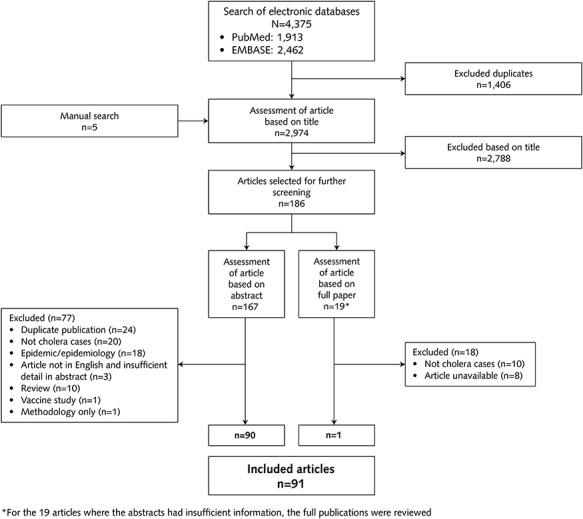
PRISMA diagram showing identification of cases for inclusion

After removal of duplicates, 2974 article titles were screened and the majority of potential papers (*n* = 2788) were excluded due to their content not relating to cholera. After further reviews of the citations at either the abstract (*n* = 167) or full publication level (*n* = 19), 91 case reports were included in this review (Figure 1). In most of the case reports, cholera vaccination information was not provided, or the authors noted that the patient had not received cholera vaccination. Seven review articles were retrieved,^22–28^ reviewing a total of 499 cases of imported cholera. Five of these articles covered 491 cases in the USA between 1965 and 2011, of which 342 were acquired outside the USA.^22^^,^^23^^,^^25^^,^^26^^,^^28^ One article covered 129 cases imported into France from 1973 to 2005^24^ and a seventh review article reported 28 cases occurring in China from 1995 to 2012, most of which were associated with eating unclean food rather than due to travel.^27^

The total number of individual cases of imported cholera specifically identified in this analysis between 1990 and 2018 was 183. Among these cases, 150 were associated with the travel of people rather than the importation of food or other miscellaneous cases.

### Cases of cholera imported from one country to another by travel

**Figure 2:**
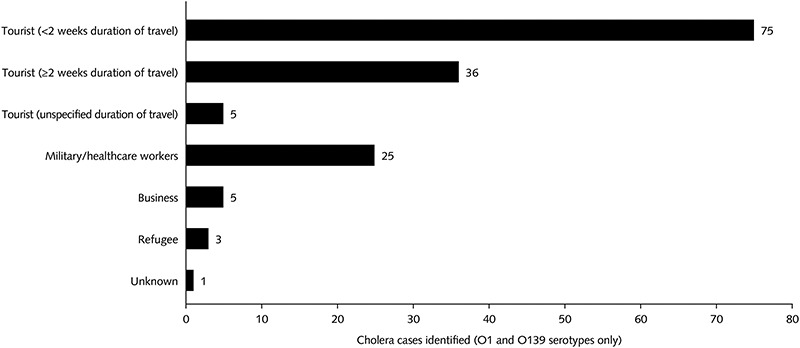
Cholera cases among different types of traveller (O1 and O139 serotype only; *n* = 150)

There were 150 cases of cholera due to serotype O1 or O130 imported as a consequence of travel. These cases are shown stratified by type of traveller in Figure 2. Most of the cases were in tourists (116 cases), followed by military personnel and/or aid workers (25 cases). The remaining reports concerned business travellers, refugees or the patient’s status was unknown. Four of these reports date from the last 5 years and approximately half date from the 1990s.

The source countries (i.e. the countries where the individual was infected) are shown in Supplementary Table 1 for serotypes O1 and O139. Infections were mostly contracted in countries and regions where cholera is endemic or in affected regions during outbreaks.^3^ South America and the Caribbean (including Haiti, Dominican Republic, Cuba, Mexico, Ecuador, and Peru) had the highest number of cases reported (*n* = 91), followed by Asia (including India, Thailand and Indonesia) with 41 cases, and Africa with 8 cases.

The case reports in the literature relating to O1 and O139 mostly concerned patients who were diagnosed in countries where cholera is not endemic, i.e. North America (79 cases). A total of 78 of these cases were imported into and diagnosed in the USA. The imported cases had mainly come from Central and South America. Among the cases diagnosed in Europe and other parts of the world, the majority of cases had originated in Asia or Africa.

### Causative serogroups

Among patients where the serogroup was identified, most cases of cholera were caused by either serogroup O1 or O139 (Table 1). Serogroup O1 was found to be responsible in 140 cases, while O139 was found to be responsible in 9 cases. There was one report of dual infection with both O1 and O139.^29^

**Table 1:** Cases caused by O1 or O139 serogroups of *Vibrio cholerae* and their region of origin

Serogroup identified	Countries of origin	Number of cases	Type of traveller	Year of diagnosis	References
	**South America and the Caribbean (91 cases)**				
Serogroup O1	Peru	32	31T, 1B	1991; 1992	[30,31]
	Ecuador	3	3VFR	1991; 1992	[32–35]
	Mexico	2	1T, 1VFR	1992; 1995	[32,36]
	Haiti	30	1VFR, 21M, 8T	2010; 2011; 2012	[37–42]
	Haiti/Dominican Republic	22	16VFR, 4M, 2B	2010	[28]
	Dominican Republic	1	1T	2011	[43]
	Cuba	1	1T	2013	[44
	**Asia (41 cases)**				
	Bangladesh	1	1T	1995	[36]
	India	16	14T, 1B	1995; 2006; 2009; 2010; 2005–2012; 2017	[36,45–50]
	Iraq	2	2T	2015	[51]
	Pakistan	4	4T	1992; 1995; 2004	[36,52,53]
	Thailand, Indonesia	10	10T	1994; 1995	[29,36,54]
	The Philippines	2	1VFR, 1B	1992; 2015	[32,35,55]
	Turkey	6	6T	2005	[56
	**Africa (8 cases)**				
	Kenya	5	5T	1995; 1998	[36,57]
	Senegal	1	1T	2005	[58]
	Tanzania	2	2R	2005	[59]
Serogroup O139	Unknown	1	NA	1994	[60]
	India	6	6T	1993; 1994	[61,62]
	Pakistan	1	1T	1994	[63]
	Thailand, Indonesia	2	2T	1994; 1995	[29,64]

Abbreviations: B, business traveller; M, military or healthcare personnel; R, refugee; T, tourist; VFR, visiting friends or relations

### Treatment of cases and outcomes

From the case reports, information on patient outcomes was not reported consistently or uniformly, making it difficult to provide summary statements of the consequence and responsiveness of therapy to medical treatments. In general, of the O1 and O139 cholera cases reviewed here, antibiotics were used in conjunction with rehydration. The average duration of illness ranged from 2 to 10 days in those cases in which this information was provided, and most patients recovered (only one death was reported among the cases reviewed here). In many of the cases, the report either did not state that antibiotics were used or, if they were, did not specify which antibiotics were used.

## Discussion

In this systematic review of the literature, the total number of individual cases of imported cholera reported between 1990 and 2018 was 183 cases—a remarkably low number, both in absolute terms and by comparison with overall data from endemic areas reported by WHO.^4^ Of these 183 cases, 150 cases of cholera were imported as a consequence of travel, and these were most often contracted in countries and regions considered high risk and where cholera is endemic, with many associated with travel to countries during outbreaks. For example, a large number of the cases reported during the 1990s were associated with travel to South America during the outbreak that began in Peru during 1991.^65^ Similarly, a considerable number of cases reported during 2010–2011 involved travel to Haiti and the Dominican Republic during the 2010 cholera outbreak.^66^ Most of the reports were of cases diagnosed in countries where cholera is not endemic, such as the USA. There were no reports of patients who were infected in one endemic country and diagnosed after travelling to another endemic country.

Most cases occurred in tourists (i.e. not business travellers or missionaries), and a majority of those had been travelling for <2 weeks. This may be because tourists outnumber other travellers to these countries. Most reported cases were caused by serogroup O1 or O139, but in a proportion of cases disease was caused by non-O1 serogroups. The proportion of non-O1 serotypes may be greater than would be expected based on epidemiologic data, which could reflect publication bias towards the reporting of uncommon serotypes.^67^ Among these cases, there were atypical presentations, and in some of these cases, the patient had underlying disease or comorbidity. However, the case reports are not consistent in terms of the level of detail that they report, making it difficult to draw firm conclusions on whether comorbidities could increase risk of infection or whether atypical presentations are related to the serogroup of the infecting organism.

The cholera cases reviewed here were mostly acquired in parts of South and Southeast Asia, Central and South America, as well as sub-Saharan Africa. The WHO records all cases reported and publishes data on endemic countries,^68^ highlighting a high risk in certain African countries, as well as Yemen and the continued risk in South Asia. The small number of cases we identified from sub-Saharan Africa may reflect the fact that fewer travellers from non-endemic countries, in whom accurate diagnosis might be possible, tend to visit areas within Africa where there is a high risk of exposure to cholera (for example, areas where there is poor sanitation and overcrowding). Alternatively, these differences may reflect under-diagnosis, under-reporting and/or publication biases. Given the challenges in estimating disease risk and incidence in travellers, future studies utilizing travel-expedient stool collection and testing methods (e.g. filter paper plus PCR) or measurement of vibriocidal antibody seroconversion associated with illness could be considered to improve estimates of traveller disease risk. Large databases such as the National Health and Nutrition Examination Survey (NHANES) or Department of Defense Serum Repository may be amenable to conduct population-based force of infection studies.^69^^,^^70^

Reported cases of cholera in travellers are rare, but since cholera can lead to severe disease, clinicians need to consider this diagnosis in any returning traveller with acute watery diarrhoea. The emergence of rapid point of care diagnostics^71^ may facilitate detection of cholera in clinics providing post-travel care for returning travellers in non-endemic countries; in areas where cholera is endemic, rapid dipstick testing is often available to test stool samples and make a diagnosis of cholera within 20 minutes.^71^ However, it should be noted that rapid dipstick testing is not confirmatory and stool culture is required to confirm the diagnosis of cholera for all suspect cases in the USA.^72^ In countries where cholera is not endemic, medical personnel may not be expecting to see it, and testing may be delayed. In our searches, we noted that several reports mentioned delays in diagnosis.^41^^,^^53^ From a pragmatic standpoint, and based on current treatment guidelines, travellers and healthcare providers should recognize the importance of initiating treatment, including rehydration and empiric antibiotics for patients during or upon return from travel with moderate to severe symptoms of watery diarrhoea that is impacting travel or activities.^17^^,^^73^ Travellers to cholera-endemic areas should be informed of the risk and of the watery diarrhoea presentation of cholera infection and given appropriate counselling and self-treatment options (oral rehydration and antibiotics for moderate to severe diarrhoea) to mitigate morbidity.

Given the effectiveness of current cholera vaccines, we support the CDC and Advisory Committee on Immunization Practices (ACIP) recommendations on the use of cholera vaccine in travellers, where vaccination is recommended for adult travellers from the USA to areas of active cholera transmission.^74^ To support these recommendations, the CDC provides an online list of countries with active cholera transmission and notes that cholera is mostly spread in limited outbreaks, with travellers rarely at risk.^1^ ACIP states that persons at higher risk for exposure might include travellers visiting friends and relatives, healthcare personnel, cholera outbreak response workers and persons traveling to or living in a cholera-affected area for extended periods. The primary prevention strategy for cholera recommended by ACIP for all travellers is consistent access to and exclusive use of safe water and food and frequent handwashing. Nonetheless, ACIP notes that travellers to areas of active cholera transmission, which include areas with current or recent endemic or epidemic cholera activity, might be exposed to toxigenic *V. cholerae* O1 through inadvertent or unexpected means, despite efforts to adhere to prevention measures.^74^ ACIP recommends that travellers who develop severe diarrhoea should seek prompt medical attention, particularly fluid replacement therapy.^74^ CVD 103-HgR is recommended for adult travellers (aged 18–64 years) from the USA to an area of active cholera transmission.^74^ An area of active cholera transmission is defined by ACIP as a province, state or other administrative subdivision within a country with endemic or epidemic cholera caused by toxigenic *V. cholerae* O1 and includes areas with cholera activity within the last year that are prone to recurrence of cholera epidemics.^74^

Several European countries, Canada and Australia have further refined these recommendations by advising the use of cholera vaccine for humanitarian aid workers in epidemic situations, individuals with underlying medical conditions such as achlorhydria that increase risk of acquiring gastrointestinal pathogens and travellers to remote areas where there is ongoing cholera transmission and limited access to safe water and medical care.^75–78^ Extension of these recommendations to other high-risk traveller populations should be considered, as additional risk data become available to guide appropriate review of evidence-based recommendations.

This review highlights the limitations of the current evidence-base surrounding cholera in travellers. We found many inconsistencies in data reporting across the articles we retrieved and in data extracted from retrospective case reports. Bearing in mind that case reports considerably underestimate true case numbers and that effective mitigation strategies are now available, we feel that better surveillance and more consistent reporting of cases would provide quantitative data capable of informing more effective preventive measures among travellers.

## Conclusions

Cholera presents a risk to those living in endemic zones and will continue to do so as long as the challenges of poverty, poor infrastructure and conflict remain. Global human development is improving generally,^79^ but counter-prevailing forces remain a serious problem in the most impoverished parts of the world. Together with factors such as increased human migration from high-risk areas, climate change and inadequate access to clean water, these conspire to extend the risk of cholera to human populations. Whether at a population or an individual level, decisions about vaccine intervention require good data to support them. This clinical review highlights the paucity of actionable information for cholera risk in travellers and identifies a number of gaps that should drive further effort to define the problem.

## Supplementary Material

Appendix_table_1_05nov19_taz085

PRISMA_2009_checklist_11NOV19_taz085
