# Supplementary material for: Cholera in travellers: a systematic review
Source: J Travel Med. 2019 Dec 4;26(8):taz085. doi: 10.1093/jtm/taz085 (PMC6927393; doi:10.1093/jtm/taz085)
Supplement: Appendix_table_1_05nov19_taz085 [file appendix_table_1_05nov19_taz085.docx]

**Appendix Table 1. Case reports of travel-related cholera contracted in one country and imported into another (O1 and O139 serotype only; n=150)**

| Country where cholera was contracted | Country where cholera was diagnosed and treated | Number and type of cases | Reference(s) |
| --- | --- | --- | --- |
| South America and the Caribbean | | | |
| Haiti, Dominican Republic | USA | 22 cases including 16 travelling to visit friends/relatives, four travelling for aid work, two travelling for business and one immigrant to the USA since epidemic began in 2010 (note: travelers could state >1 reason for travel) | 28 |
| Dominican Republic | USA | 1 tourist | 43 |
| Haiti | USA | 1 tourist | 40 |
|  |  | 1 tourist | 41 |
|  |  | 5 tourists | 39 |
|  |  | 1 tourist, bariatric surgery possibly a factor | 42 |
| Haiti | Canada | 1 tourist attending family funeral | 37 |
| Haiti | Martinique (France) | 21 French military policemen and healthcare workers | 38 |
| Cuba | Italy | 1 tourist | 44 |
| Peru | USA | 31 tourists (airline passengers). Serotype O1 confirmed in 5 cases | 30, 80-82 |
|  |  | 1 doctor returning from a conference | 31 |
| Asia | | | |
| India | USA | 1 tourist with serogroup O139 | 62 |
|  |  | 1 tourist, cholera associated with mesenteric panniculitis | 83 |
| India | France | 2 tourists | 47 |
| India | South Africa | 1 tourist | 45 |
| India | Russia | 6 cases, including 5 tourists and 1 business traveler | 46 |
| India | Romania | 1 tourist | 48 |
| India | Singapore | 5 tourists (0139 serotype) | 61 |
| India | France | 1 tourist | 50 |
| India | The Netherlands | 1 tourist (child) | 49 |
| Turkey | Belgium | 6 tourists (2 confirmed, 4 probable) | 56 |
| Indonesia | Taiwan | 1 tourist | 84 |
| Iraq | Kuwait | 2 tourists; genetic analysis | 51 |
| The Philippines | Germany | 1 business traveler | 55 |
| Pakistan | USA | 2 tourists (both children) | 52, 53 |
| Pakistan | Germany | 1 tourist | 63 |
| Thailand | UK and USA | 6 tourists on a cruise ship, who acquired the infection from food | 85 |
| Thailand | Slovenia | 1 tourist | 54 |
| Thailand | USA | 1 tourist, O139 serogroup | 64 |
| Thailand, Indonesia | Japan | 1 tourist, co-infection with O1 and O139 | 29 |
| Africa | | | |
| Tanzania | Australia | 2 refugees | 59 |
| Senegal | Italy | 1 tourist | 58 |
| Kenya | UK | 4 tourists | 57 |
| Reports with multiple countries | | | |
| Unknown | Denmark | 1 Thai woman, source unknown | 60 |
| Ecuador, The Philippines, Mexico | USA | 5 individual cases returning from native countries (tourists) | 32, 35 |
| India, Bangladesh, Indonesia, Pakistan, Thailand, Kenya, Mexico | UK | 10 individual cases (tourists) | 36 |
